# Evidence-based medical equipment management: a convenient implementation

**DOI:** 10.1007/s11517-019-02021-x

**Published:** 2019-08-10

**Authors:** Ernesto Iadanza, Valentina Gonnelli, Francesca Satta, Monica Gherardelli

**Affiliations:** 1 Information Engineering Department, University of Florence, Via S. Marta, 3, 50139 Florence, Italy; 2 ESTAR - Dipartimento Tecnologie Informatiche e Sanitarie UOC, Tecnologie Sanitarie AOU Careggi/Meyer, Largo Brambilla 3, 50141 Florence, Italy

**Keywords:** Evidence-based maintenance, Health technology management, Key performance indicators, Medical equipment, Clinical engineering

## Abstract

Maintenance is a crucial subject in medical equipment life cycle management. Evidence-based maintenance consists of the continuous performance monitoring of equipment, starting from the evidence—the current state in terms of failure history—and improvement of its effectiveness by making the required changes. This process is very important for optimizing the use and allocation of the available resources by clinical engineering departments. Medical equipment maintenance is composed of two basic activities: scheduled maintenance and corrective maintenance. Both are needed for the management of the entire set of medical equipment in a hospital. Because the classification of maintenance service work orders reveals specific issues related to frequent problems and failures, specific codes have been applied to classify the corrective and scheduled maintenance work orders at Careggi University Hospital (Florence, Italy). In this study, a novel set of key performance indicators is also proposed for evaluating medical equipment maintenance performance. The purpose of this research is to combine these two evidence-based methods to assess every aspect of the maintenance process and provide an objective and standardized approach that will support and enhance clinical engineering activities. Starting from the evidence (i.e. failures), the results show that the combination of these two methods can provide a periodical cross-analysis of maintenance performance that indicates the most appropriate procedures.

**Graphical abstract:**
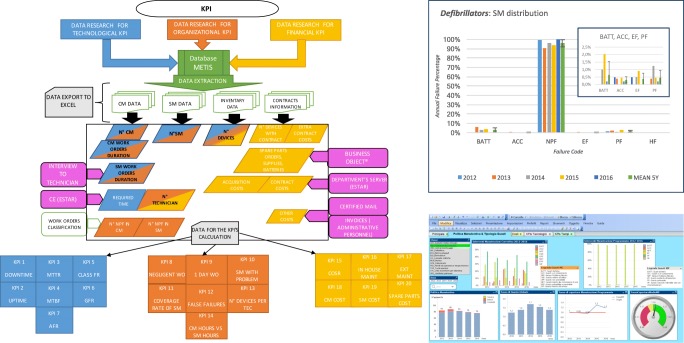
The left side shows a block diagram of the process needed to calculate the proposed set of KPIs, starting from technological, organizational and financial data. On the upper right it is shown an example of scheduled maintenance analysis for a specific class of equipment (legend in the article body). The bottom right part shows how the KPIs can be implemented in a business intelligence dashboard.

The left side shows a block diagram of the process needed to calculate the proposed set of KPIs, starting from technological, organizational and financial data. On the upper right it is shown an example of scheduled maintenance analysis for a specific class of equipment (legend in the article body). The bottom right part shows how the KPIs can be implemented in a business intelligence dashboard.

## Introduction

Today’s rapid and continuous technological evolution, which affects most production sectors, also involves healthcare. Indeed, healthcare technologies have become an essential part of the provided services, as they play increasingly significant roles in the diagnosis and treatment of patients.

The complexity of the technological assets found in healthcare facilities, in terms of number and diversity, is reflected in the complexity of technology management, which must be efficient so that the equipment can always be used safely and appropriately. From this perspective, maintenance is a key process throughout the life cycle of every medical device. Maintenance planning requires the assessment of a number of parameters, including how a piece of equipment is used, how often it is used, its intended use, risk associated with its usage and its failure rates.

There are two main types of maintenance required for medical equipment in all hospitals: scheduled maintenance (SM) and corrective maintenance (CM). SM, in compliance with the manufacturer’s instructions, includes the operations performed at scheduled times to reduce deterioration from use (often referred to as “preventive maintenance”) or the occurrence of functional failures. CM comprises the repair of the equipment’s functions (i.e. its restoration) as well as its replacement when repair is not feasible due to costs or obsolescence [15].

Maintenance is also a crucial aspect of the activities in a hospital’s clinical engineering (CE) department because it involves significant human and financial resources. Therefore, the assessment of the effectiveness of any maintenance programmes is strictly linked to the optimization of the use of available resources in CE departments [20].

This is the context of this research work, which shows an evidence-based approach to monitoring maintenance performance in a highly complex hospital with vast and varied technology. Although the expression “evidence-based” is well known in the medical literature, it may also be applied to maintenance. Evidence-based maintenance (EBM) begins with the analysis of evidence (i.e. failures) to monitor the maintenance effectiveness and plan any necessary changes to improve it. Maintenance reports in most hospitals describe only the failures, the repair procedures and any spare parts used. What these reports never provide is information about any measures needed to prevent that failure [21]. Knowledge of the history of a failure enables the monitoring and improvement of the current maintenance strategy so that the most appropriate approach can be found. Ultimately, when the effectiveness, reliability and availability of medical equipment are improved through maintenance, the safety of staff and patients is improved.

The objective of this study is to verify the feasibility of implementing an evidence-based method (i.e. based on the history of failures) for maintenance. In this way, through the study of current maintenance procedures, the steps required for strategic maintenance policy changes can be applied. This research paper is grounded on the EBM approach applied by Wang et al. [21–24].

The first step of this process was to classify the maintenance work orders (WOs) using a set of codes. The same small set of codes selected in [21] was used to standardize and simplify WO classification. Then, analysis of the SM and CM medical equipment records enabled the identification of unusually high code incidence and issues related to possible omissions.

The second step was the design of a novel set of key performance indicators (KPIs) useful for assessing the performance of medical equipment maintenance. The most suitable indicators for the available data, information and context were selected among those available in the literature.

Some prior papers related to the EBM approach and to the use of KPIs for evaluating medical equipment maintenance performance were presented by the authors at international conferences [10–12, 17] or published in international journals [2].

## Materials and methods

This study began in December 2016 at Careggi University Hospital, which has 1367 beds and 16,209 pieces of medical equipment. Management of the medical equipment and its maintenance is entrusted to the Department of Information and Health Technology of the regional health service body ESTAR (Ente di Supporto Tecnico-Amministrativo Regionale), which involves 6 engineers, 5 technicians, 2 administrative staff units, and the head of the service.

A mixed maintenance strategy is in place due to the technological complexity and the number and type of different pieces of equipment. Dedicated internal technicians take care of scheduled and corrective maintenance of some classes of equipment and are in charge of first-level maintenance in partner agreements with manufacturers or distributors. Internal maintenance is adopted for surgical lamps (LSC), ceiling-mounted units (PSO) and telemetry devices (UTC).

For critical or high-tech devices, maintenance is covered by full risk agreements with manufacturers or authorized service centres. External maintenance is adopted for anaesthesia machines (ANS), central monitoring systems (CMO), electrocardiographs (ECG), vital parameter monitors (MON), surgical tables (TOP) and ventilators (VPO).

The maintenance of aspirators (ACH), defibrillators (DEF), electro-surgery units (ELB) and oximeters (OOR) is entrusted to a global service provider also in consideration of their amount and the diversity of their manufacturers.

The analysis in this paper concerns data from the equipment used in intensive care and surgery departments, including vascular intervention. Table 1 shows the number of operating rooms involved in the analysis and the number of intensive care beds. Indeed, these departments, among the most critical in the hospital, are characterized by high technological heterogeneity. The data refer to the period 2012–2016.

**Table 1:** Analysed data

Departments	Rooms			Beds	Wards	
Operating rooms		40			9	
Interventional		5			5	
Intensive care				165	10	
Device type	TOT Units (U&oU)	TOT CM WO	TOT	OR&IC	OR&IC	OR&IC SM WO
			SM WO	Units	CM WO	
Anaesthesia Machine	162	802	491	109	593	444
Aspirator	377	160	287	43	20	42
Ceiling mounted unit	319	284	522	214	165	386
Central monitoring	63	212	147	33	114	87
Defibrillator	410	1463	2036	128	438	709
Electrocardiograph	356	1384	947	57	155	148
Electrosurgical	205	287	408	148	181	342
Monitor	900	1294	3337	487	547	1794
Oximeter	613	557	1120	154	110	297
Surgical lamp	354	411	1222	225	239	987
Surgical table	93	520	382	70	349	211
Telemetry	104	99	142	27	59	51
Ventilator	203	796	831	155	611	748
Total analysed data	4159	8269	11,872	1850	3581	6246

**Fig. 1:**
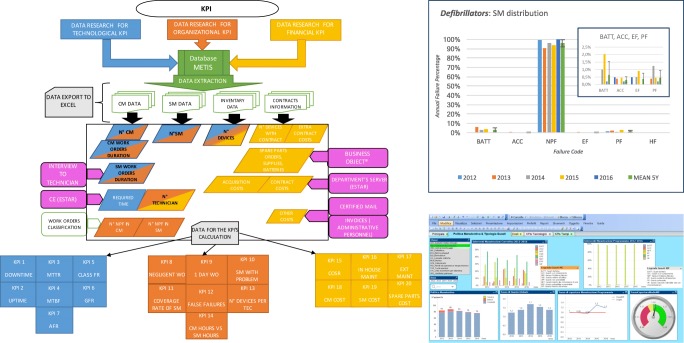
The left side shows a block diagram of the process needed to calculate the proposed set of KPIs, starting from technological, organizational and financial data. On the upper right, an example of scheduled maintenance analysis for a specific class of equipment is shown (legend in the article body). The bottom right part shows how the KPIs can be implemented in a business intelligence dashboard

Figure 1 describes, in a block diagram, the process for calculating the proposed set of KPIs, as detailed in this section. The process starts with research on data for technological, organizational and financial KPIs that can be found in the medical equipment database. Then, all the required data for CM, SM, inventory and information from maintenance contracts are collected. Information concerning factors such as costs, durations, number of devices and human resources is meticulously selected also from server software, certified mail and invoices as well as from direct interviews with technicians. The KPIs can be calculated and analysed graphically and with the help of business intelligence software.

### Data analysis of hospital equipment

The first step was to identify the classes of equipment as a target for the focused research analysis.

It was decided to give relevance to the most numerous and critical devices. The choice was restricted to classes of equipment with more than 40 units belonging to the two aforementioned departments. Table 1 shows the 13 selected classes with the device type in the first column and the quantity in the second. We analysed technical reports on CM and SM activities, including preventive maintenance, electrical safety tests and quality control. Table 1 also shows the number of CM and SM WOs in the third and fourth columns. The rightmost three columns show how many pieces of equipment are in use in surgery departments and intensive care units, as well as the related CM and SM work orders.

A total of 14.06% of the data were excluded from the analysis because the related reports lacked enough information for a proper classification.

### Failure classification

The purpose of classifying the maintenance operations was to analyse and monitor the types of performed operations. The number of CM cases corresponds to the number of failures that occurred (except for false failures, NPF). The same codes used in [21] are used to identify each failure type summarized in Table 2. Each individual CM and SM technical report was carefully analysed so that the many failures that occur each year could be catalogued. In ambiguous cases, when there was a possible correspondence of two or more codes with the same failure, the most appropriate code was selected through careful analysis performed in cooperation with CE technicians and staff.

**Table 2:** Failure codes

Code	Description	CM/SM
NPF	No problem found	Both
BATT	Battery failure	Both
ACC	Accessory failure (including supplies)	Both
NET	Failure related to network	CM
USE	Failure induced by use (i.e. abuse, accident, environment conditions)	CM
UPF	Unpreventable failure caused by normal wear and tear	CM
PPF	Predictable and preventable failure	CM
SIF	Induced by service (i.e. caused by a technical intervention not properly completed or premature failures of a part just replaced)	CM
EF	Evident failure (i.e. evident to user but not reported)	SM
PF	Potential failure (i.e. in process of occurring)	SM
HF	Hidden failure (i.e. not detectable by the user unless special test or measurement equipment)	SM

### KPIs

The UNI EN 15341:2007 standard [19] describes a system for managing KPIs to measure maintenance performance as influenced by key maintenance factors and to assess and improve efficiency and effectiveness. The standard is applicable to many industrial and technical sectors. The maintenance of medical devices must ensure equipment availability and reliability (linked to the safety of the device).

The standard suggests that the KPIs be structured into three groups to measure every aspect of the maintenance process. A thorough review of the literature led to the selection of the three groups below to match the CE department’s data and requirements. These KPIs are as follows:

1. Financial, with the assessment of the cost-effectiveness of the performance being the primary objective [1, 3, 6, 19]
2. Technological, with the assessment of the operational performance of the equipment in terms of its reliability and availability (related to customer satisfaction) as its aim [1, 13, 16, 17, 19, 25, 27]
3. Organizational, which is related to internal processes and staff productivity [1, 5, 10, 13, 14, 16, 17, 25, 27]

Taking into account the criteria suggested by the above standard, as well as the analysed literature and our personal knowledge and needs from the field, we designed a set of 20 KPIs, which are thoroughly described in Table 3 below. The table summarizes the information on the chosen indicators: their name, the type of indicator (financial, organizational or technological), the mathematical definition and the rationale behind it. Moreover, the table indicates which activities are pertinent to each indicator, between CM and SM. Internal maintenance (IM) and/or external maintenance (EM) activities are indicated for each indicator as well. The identified indicators were calculated for each year from 2012 to 2016 for each of the 13 chosen equipment classes to obtain the overall behaviour and evolution of each indicator over time.

**Table 3:** Key performance indicators

Index	KPI type	Definition	Rationale	Involved activity			
				Corrective Maintenance CM	Scheduled Maintenance SM	Internal Maintenance IM	External Maintenance EM
KPI 1 Downtime (%) (non-availability time)	**T**	${T}_{\mathrm{down}}\left(\%\right)=\frac{T_{\mathrm{nd}}}{RT}100$ with: *T*_nd_ = non-availability time per year; *RT* = *Required Time* per year.	Operational efficiency, actual equipment availability compared with requirements.	X	X		
KPI 2 Uptime (%) (availability time)	**T**	${T}_{\mathrm{up}}\left(\%\right)=\frac{T_d}{RT}100$ with: *T*_*d*_ *= RT − T*_nd_	Operational efficiency, actual equipment availability compared with requirements.	X			
KPI 3 MTTR (mean time to restoration)	**T**	$\mathrm{MTTR}=\frac{T_f}{N_{CM}}$ *T*_*f*_ is the off-duty time for failure; *N*_CM_ is the total number of corrective actions.	Parameter of reliability, availability.	X			
KPI 4 MTBF (mean time between failures)	**T**	$\mathrm{MTBF}=\frac{T_d}{N_{CM}}$ *T*_*d*_ is the availability time; *N*_*CM*_ is the total number of corrective actions.	Parameter of reliability, availability.	X			
KPI 5 Class failure ratio (fails per class)	**T**	$Class\kern0.17em Failure\kern0.17em Ratio=\frac{N_{CM i}}{N_{CM}}$ *N*_*CMi*_ is the number of corrective actions per year applied to the *i*th equipment class; *N*_*CM*_ is the total number of corrective actions in the same year.	Failure rate of each class of equipment	X			
KPI 6 Global failure rate (defectiveness)	**T**	${G}_{\mathrm{FR}}=\frac{N_{CM}}{N_{\mathrm{dev}}}$ *N*_*CM*_ is the total number of corrective actions per year; *N*_dev_ is the number of devices in the inventory at the end of the year.	Fault occurrences related to the number of devices	X			
KPI 7 AFR: age failure rate	**T**	$\mathrm{AFR}=\frac{{\left.{N}_{CM}\right|}_{\mathrm{age}\;\mathrm{class}}}{{\left.{N}_{\mathrm{dev}}\right|}_{\mathrm{age}\;\mathrm{class}}}$ *N*_*CM*_ is the total number of corrective actions per year; *N*_dev_ is the device number. Age classes: 0–2 years, 3–5 years, 6–9 years, ≥10 years	Device obsolescence	X			
KPI 8 “Negligent” actions (%)	**O**	$\mathrm{Negligent}\kern0.17em \mathrm{Actions}\left(\%\right)=\left(\frac{N_{\mathrm{negl}}}{N_{CM}}\right)100$*N*_negl_ is the number of corrective actions per year, that have not been completed within 30 days (“negligent” actions); *N*_*CM*_ is the number of corrective actions per year.	Operational performance of maintenance process	X			
KPI 9 “1 day” actions	**O**	$1\mathrm{day}\;\mathrm{actions}\left(\%\right)=\left(\frac{N_{1\mathrm{day}}}{N_{CM}}\right)100$ *N*_1day_ is the number of corrective actions per year, that have been completed within 24 h; *N*_CM_ is the number of corrective actions per year.	Operational performance of maintenance process	X			
KPI 10 SM with failure (%)	**O**	$\mathrm{SM}\;\mathrm{with}\kern0.17em \mathrm{failure}\left(\%\right)=\left(\frac{N_{\mathrm{SM}\;\mathrm{failure}}}{N_{SM}}\right)100$ *N*_SM failure_ is the number of scheduled maintenance actions per year with code ≠ NPF; *N*_SM_ is the number of scheduled maintenance actions per year.	Scheduled maintenance intervention with fault occurred		X		
KPI 11 SM coverage rate (scheduled maintenance)	**O**	$\mathrm{SM}\;\mathrm{Coverage}\kern0.17em \mathrm{Rate}\;\left(\%\right)=\left(\frac{N_{SM}}{N_{\mathrm{dev}}}\right)100$ SM coverage rate *N*_*SM*_ is the number of scheduled actions per year; *N*_dev_ is the number of devices available in that year.	Scheduled Maintenance conformity to the requirements		X		
KPI 12 No problem found (fake faults) (%)	**O**	$N{}^{\circ} NPF\left(\%\right)=\left(\frac{N{}^{\circ} NPF}{N_{CM}}\right)100$ *N*_*CM*_ is the number of corrective actions per year.	No fault found during the corrective maintenance work order	X			
KPI 13 No. devices per technician (internal)	**O**	$\left(\frac{\mathrm{No}.\mathrm{device}}{\mathrm{No}.\mathrm{technicians}}\right)$	Maintenance workload	X	X		
KPI 14 *Time cost of the workforce*	**O**	Working hours spent on corrective maintenance vs working hours spent on scheduled maintenance	Maintenance-workload comparison between corrective and scheduled maintenance	X	X		
KPI 15 COSR (cost of service ratio *=* global maintenance to acquisition cost) (%)	**F**	$\mathrm{COSR}\left(\%\right)=\left(\frac{\mathrm{Global}\kern0.17em \mathrm{Maintenance}\kern0.17em \mathrm{Cost}}{\mathrm{Acquisition}\kern0.17em \mathrm{Cost}}\right)100$	Maintenance service: financial performance (cost-effectiveness).	X	X	X	X
KPI 16 External maintenance Cost (% with respect to total maintenance cost)	**F**	$\left(\frac{\mathrm{External}\kern0.17em \mathrm{Maintenance}\kern0.17em \mathrm{Cost}}{\mathrm{Total}\kern0.17em \mathrm{Maintenance}\kern0.17em \mathrm{Cost}}\right)100$ where external maintenance cost = scheduled and corrective external maintenance costs	Impact of external maintenance on the total cost of the maintenance service				X
KPI 17 Internal maintenance cost (% with respect to total maintenance cost)	**F**	$\left(\frac{\mathrm{Internal}\kern0.17em \mathrm{Maintenance}\kern0.17em \mathrm{Cost}}{\mathrm{Total}\kern0.17em \mathrm{Maintenance}\kern0.17em \mathrm{Cost}}\right)100$ where internal maintenance cost = scheduled and corrective internal maintenance costs	Impact of internal maintenance on the total cost of the maintenance service			X	
KPI 18 Corrective maintenance cost (CM) (% with respect to total maintenance cost)	**F**	$\left(\frac{\mathrm{Corrective}\kern0.17em \mathrm{Maintenance}\kern0.17em \mathrm{Cost}}{\mathrm{Total}\kern0.17em \mathrm{Maintenance}\kern0.17em \mathrm{Cost}}\right)100$ where corrective maintenance cost = internal CM cost + external CM cost	Maintenance type: impact of corrective maintenance on the total cost of the maintenance service.	X			
KPI 19 Scheduled maintenance cost (SM) (% with respect to total maintenance cost)	**F**	$\left(\frac{\mathrm{Scheduled}\kern0.17em \mathrm{Maintenance}\kern0.17em \mathrm{Cost}}{\mathrm{Total}\kern0.17em \mathrm{Maintenance}\kern0.17em \mathrm{Cost}}\right)100$where scheduled maintenance cost = internal SM cost + external SM cost	Maintenance type: impact of scheduled maintenance on the total cost of the maintenance service.		X		
KPI 20 Cost of spare parts (+ consumables) (% with respect to total maintenance cost)	**F**	$\left(\frac{\mathrm{Cost}\kern0.17em \mathrm{of}\kern0.17em \mathrm{Spare}\kern0.17em \mathrm{Parts}}{\mathrm{Total}\kern0.17em \mathrm{Maintenance}\kern0.17em \mathrm{Cost}}\right)100$	Maintenance: Spare Parts and consumables.	X	X	X	X

The set of 20 indicators in Table 3 not only come from the UNI EN 15341 standard but also are intended as a novel research result of our study.

To further clarify the indicator concepts of *downtime* and *uptime*, the European standard EN 13306:2010 was used as a reference [18]. Downtime is the time interval throughout which an item is not capable of performing its function. Uptime is the time interval throughout which an item is fully functional. The well-known *mean time to restoration* (MTTR) and *mean time between failures* (MTBF) are the average times to restoration of function and the average time between consecutive failures, respectively.

With regard to the financial indicators, the acquisition cost (used in KPI-15) was derived from the tables showing the purchase value estimates for each equipment class supplied by the CE department in 2014, increased by 20%. Furthermore, a 2% increase or decrease was estimated for the years subsequent to and prior to 2014, respectively. The acquisition costs reached for each class were multiplied by the annual number of each class. The decision to use purchase value estimates was justified, as indicated in the literature [8], by the fact that the purchase cost of each individual device represents only the initial portion of the total cost of ownership of that device. The total cost of ownership comprises several cost items, such as contracts, spare parts, accessories, consumables and instruments used to perform test measurements.

To calculate total maintenance costs, only the cost items clearly linked to each device were considered. Therefore, the total maintenance cost was calculated using these cost items:

- Contract costs
- Costs for spare parts and consumables
- Costs for batteries
- Costs for internal maintenance personnel
- Extra-contractual costs (all costs not elsewhere covered)

## Results

This section displays the analysis and the graphs derived from the methods described in the previous section. Given the large amount of data, only the graphs related to some of the devices analysed are included. The histograms show a stable pattern when more than 50 sets of data are used, which is in line with the literature [21]. By analysing all the equipment classes used in the hospital, a characteristic performance shape may be obtained for this specific hospital, which can then be used to make comparisons/benchmarks with other hospitals.

### Distribution of classified failures

**Fig. 2:**
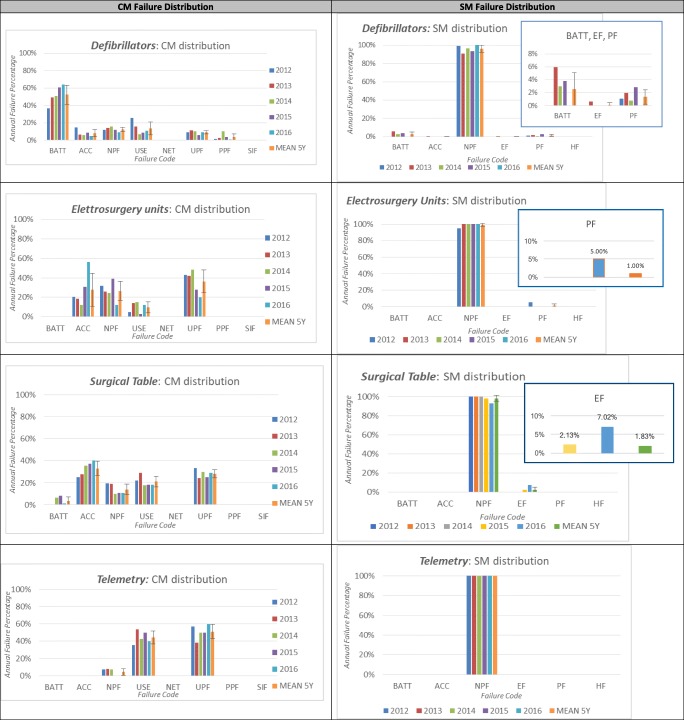
CM and SM failure distributions related to surgical tables, telemetry equipment, electro-surgery units and defibrillators in different years of the considered period

The histograms in Fig. 2 show the distribution of failure codes obtained from CM and SM WOs, which were related to surgical tables, telemetry equipment, electro-surgery units and defibrillators. The graphs obtained through the analysis of CM WOs are in the first column. The graphs from SM WOs are in the second column. Details are sometimes shown for a better data comprehension.

The first five histogram bars for each type of failure represent the five investigated years (2012 to 2016). The rightmost one is the average value with error bars of ± 1 standard deviation (SD). The height of the bars represents the percentage of failures found in CM or SM WOs.

The CM values were corrected using the equipment type failure rate (ETFR), which is the percentage of units within a specific equipment type that failed each year [21]. This correction is necessary when a combination of CM and SM failure code distributions is required to provide a more complete view of the equipment fault history.

In fact, the CM WOs are only related to failed units and did not consider units that had not failed. Instead, the SM WOs refer to all units.

From the top left chart in Fig. 2 (CM distribution for defibrillators), some interesting information can be gleaned about the effectiveness of the most common types of maintenance procedures used in the category of defibrillators. It appears that the most significant failure category involves batteries (BATT). Specifically, in 2015, 60.40% of CM WOs were for battery failures, 8.91% were related to wear and 5.94% were unpreventable failures. In 2016, 93.33% of the SM WOs were NPF, 3.81% were for BATT and 2.86% were preventable failures (PF).

From a review of the literature [23], a comparison of maintenance strategies in different hospitals shows consistent differences. As expected, BATT failures were lower in cases where scheduled maintenance was performed more frequently. The paper confirms that a higher frequency of scheduled maintenance reduces certain types of failures (BATT). Therefore, the pattern from our case could be explained by a lower rate of scheduled maintenance coverage than the rate provided by the maintenance plan before 2016.

Another interesting fact emerged by comparing the CM and SM fault patterns for defibrillators (Fig. 2), the latter including preventive maintenance, electrical safety audits and quality control. The classes with potential maintenance omissions are PF and EF in SM and PPF in CM. The latter category is responsible for approximately 3% of all failures. Through the analysis of the technical reports, this circumstance may be ascribed to poor first-level maintenance by health personnel. This result is in line with the peculiarity of Careggi University Hospital, which is an extremely large healthcare facility with a very high rate of personnel turnover. This could affect staff accountability in asset management (the conflict between university and hospital property) reflected in the consistency of the USE failure class incidents that include accidental failures and failures due to the misuse of equipment. These data confirm the importance of staff training. Since there is no user-training programme beyond initial training during device testing, there is a high incidence of failures due to improper device management.

From the corrective maintenance pattern for electro-surgery units (Fig. 2), it is evident that the most affected category in this case is UPF. This type includes a broader range of failures, normally attributed to wear. The scheduled maintenance pattern shows a prevalence of procedures with positive outcomes (NPF). Nonetheless, there is a 1% potential failure rate, which might be related to issues that can be resolved by increasing the frequency of scheduled maintenance and by paying more attention to checking the correct function of components that are more prone to failure (pedal, plates and handpieces).

By analysing the CM procedures on surgical tables from 2012 to 2016 (Fig. 2), the most significant category of failures is ACC (failures of accessories). Some of the indirect actions the CE department could implement to reduce this type of failure belong to the procurement stage. Giving importance to the reliability of accessories and spare parts as well as analysing the brands in use with a higher failure rate in relation to the total number of units in the inventory could be effective in reducing these failures. The scheduled maintenance chart clearly shows that, every year, scheduled maintenance procedures on surgical tables take place with no negative results reported. The remaining 2% of the maintenance WOs were coded as evident failures (EF). To reduce this type of failure, personnel should be trained to immediately report failures and problems that are evident and can be identified with no special tools or measurements.

The telemetry corrective maintenance histogram for failures from 2012 to 2016 shows a failure history that includes three types of problems: UPF, USE and NPF. The peak of the UPF category for 2016 is in line with the list published by ECRI for the “Top 10 Health Technology Hazards” for 2016 [9]. This report states that telemetry failures and the resulting lack of monitoring of a patient’s vital signs are in 4th place among the hazards to patients from medical technology failures. This report mainly discusses the improper use of medical devices. Moreover, it highlights the importance of taking actions focused on reducing USE because this is the other category of failures that affects these devices. For example, among the indirect actions the CE department could implement, it is worth reiterating that better staff training can be effective in appropriate telemetry management.

From a review of the technical reports on telemetry, the high level of stress on these devices emerges, as they are constantly connected to patients being monitored. The purchase of more robust and reliable equipment that is able to better handle high technological stress levels could be a solution. The scheduled maintenance procedures on these devices have a 100% success rate with all positive outcomes (NPF).

### Assessment using KPIs

This section presents some considerations on the values of the set of indicators identified in Section 2.3.

All the calculated KPIs for each equipment class, as well as the distribution of the equipment in classes of age, are shown in Tables 4, 5, 6a and b and 7 below.

**Table 4:** Technical KPIs

Equipment class	Technical KPIs									
	KPI 1 Downtime (%) (without negligent work orders)	KPI 2 Uptime (%) (without negligent work orders)	KPI 3 MTTR (without negligent work orders)	KPI 4 MTBF (without negligent work orders)	KPI 5 Class failure ratio	KPI 6 Global failure rate	KPI 7 AFR: age failure rate Mean 5Y ± SD Age class			
	Mean 5Y ± SD	Mean 5Y ± SD	(days)	(months)	Mean 5Y ± SD	Mean 5Y ± SD	0–2 years	2–5 years	5–10 years	> 10 years
CMO	0.62% ± 0.36%	99.38% ± 0.36%	2.68	14.10	3.13 ± 0.89	0.87 ± 0.26	0.5 ± 0.5	0.7 ± 0.2	1.3 ± 0.4	0.1 ± 0.2
TOP	2.74% ± 0.69%	97.26% ± 0.69%	4.70	1.91	9.59 ± 2.52	1.35 ± 0.36	1.0 ± 0.4	1.9 ± 1.3	3.2 ± 1.0	1.3 ± 0.3
UTC	0.52% ± 0.27%	99.48% ± 0.27%	3.05	15.26	1.67 ± 0.48	0.64 ± 0.12	0.0 ± 0.0	0.6 ± 0.6	0.3 ± 0.4	0.0 ± 0.0
ANS	2.14% ± 0.61%	97.86% ± 0.61%	3.02	1.74	16.59 ± 1.92	1.45 ± 0.18	1.2 ± 0.7	1.9 ± 0.7	1.9 ± 0.5	1.1 ± 0.2
ELB	0.49% ± 0.30%	99.51% ± 0.30%	2.59	6.22	5.14 ± 1.49	0.41 ± 0.07	0.2 ± 0.1	0.4 ± 0.1	0.6 ± 0.2	0.8 ± 0.5
VPO	0.98% ± 0.4%	99.02% ± 0.40%	3.09	9.04	17.10 ± 1.83	1.07 ± 0.12	0.6 ± 0.4	1.4 ± 0.8	1.8 ± 0.5	1.1 ± 0.1
ACH	0.13% ± 0.16%	99.87% ± 0.16%	2.30	20.38	0.58 ± 0.42	0.12 ± 0.06	0.2 ± 0.2	0.2 ± 0.3	0.0 ± 0.1	0.2 ± 0.2
ECG	0.54% ± 0.33%	99.46% ± 0.33%	2.85	11.74	4.37 ± 1.54	0.53 ± 0.16	0.9 ± 0.5	0.9 ± 0.9	0.5 ± 0.3	0.5 ± 0.1
LSC	0.64% ± 0.44%	99.36% ± 0.44%	4.24	8.81	6.63 ± 2.54	0.30 ± 0.13	0.0 ± 0.1	0.5 ± 0.4	0.3 ± 0.1	0.5 ± 0.1
DEF	0.47% ± 0.36%	99.53% ± 0.36%	1.71	12.63	12.19 ± 2.30	0.96 ± 0.25	0.2 ± 0.1	0.4 ± 0.4	1.3 ± 0.3	1.2 ± 0.5
PSO	0.21% ± 0.12%	99.79% ± 0.12%	2.41	15.43	4.55 ± 1.59	0.16 ± 0.05	0.1 ± 0.1	0.3 ± 0.2	0.1 ± 0.1	0.2 ± 0.0
OOR	0.13% ± 0.08%	99.87% ± 0.08%	1.83	34.56	3.13 ± 1.18	0.20 ± 0.06	0.1 ± 0.1	0.3 ± 0.1	0.2 ± 0.1	0.1 ± 0.1
MON	0.31% ± 0.05%	99.69% ± 0.05%	3.73	38.04	15.33 ± 1.21	0.29 ± 0.04	0.1 ± 0.1	0.7 ± 0.6	0.5 ± 0.1	0.3 ± 0.1

**Table 5:** Distribution of the equipment in age classes

Age classes (mean no. of units for each age class) Mean 5Y ± SD				
Equipment class	0–2 years	2–5 years	5–10 years	> 10 years
CMO	28.49% ± 16.48%	31.28% ± 20.84%	31.33% ± 13.90%	8.90% ± 4.22%
TOP	32.77% ± 12.76%	13.64% ± 14.79%	11.64% ± 2.86%	41.94% ± 5.55%
UTC	0.00% ± 0.00%	38.00% ± 35.64%	38.74% ± 45.63%	23.26% ± 13.10%
ANS	26.43% ± 14.49%	25.33% ± 6.98%	19.32% ± 3.48%	28.92% ±6.77%
ELB	24.74% ± 3.72%	24.51% ± 4.55%	19.12% ± 5.58%	31.63% ± 4.11%
VPO	10.78% ± 5.63%	23.37% ± 18.27%	23.93% ± 11.09%	41.92% ± 3.14%
ACH	9.95% ± 6.01%	5.13% ± 5.40%	15.17% ± 6.93%	69.75% ± 6.53%
ECG	9.96% ± 8.23%	10.05% ± 7.45%	21.03% ± 3.26%	58.96% ± 6.66%
LSC	33.97% ± 21.25%	20.38% ± 20.73%	12.75% ± 5.17%	32.90% ± 3.21%
DEF	10.63% ± 4.91%	13.72% ± 8.91%	33.67% ± 4.65%	41.98% ± 6.04%
PSO	42.34% ± 29.29%	25.64% ± 28.00%	10.01% ± 2.47%	22.00% ± 0.90%
OOR	23.38% ± 9.96%	28.21% ± 12.41%	27.20% ± 9.96%	21.21% ± 5.59%
MON	33.02% ± 20.56%	25.81% ± 17.47%	22.01% ± 5.57%	19.16% ± 2.93%

**Table 6:** Organizational KPIs

	Organizational KPI				
**a**					
**Equipment class**	**KPI 8** “Negligent” actions (%)	**KPI 9** “1 day” actions	**KPI 10** SM with failure (%)	**KPI 11** SM coverage rate	**KPI 12** No problem found (%)
	Mean 5Y ± SD	Mean 5Y ± SD	Mean 5Y ± SD	Mean 5Y ± SD	Mean 5Y ± SD
CMO	7.51% ± 10.52%	42.25 ± 17.41	1.00% ± 2.24%	0.67 ± 0.07	16.97% ± 5.73%
TOP	10.64% ± 6.63%	22.35 ± 5.97	1.83% ± 3.04%	0.84 ± 0.30	14.05% ± 4.73%
UTC	55.63% ± 12.48%	9.14 ± 11.72	0.00% ± 0.00%	0.56 ± 0.31	4.40% ± 4.02%
ANS	4.39% ± 4.84%	45.05 ± 9.19	1.82% ± 1.31%	1.09 ± 0.15	26.12% ± 4.93%
ELB	5.25% ± 4.21%	45.83 ± 24.69	1.00% ± 2.24%	0.77 ± 0.15	26.51% ± 9.97%
VPO	7.35% ± 3.74%	49.65 ± 11.27	0.87% ± 0.84%	1.31 ± 0.21	20.18% ± 2.92%
ACH	0.00% ± 0.00%	58.83 ± 25.71	0.00% ± 0.00%	0.30 ± 0.26	38.00% ± 41.47%
ECG	2.78% ± 2.89%	47.23 ± 19.52	0.83% ± 1.86%	0.51 ± 0.25	19.85% ± 6.42%
LSC	4.21% ± 1.39%	51.61 ± 15.00	1.54% ± 1.84%	1.18 ± 0.51	13.03% ± 9.50%
DEF	3.81% ± 2.93%	64.49 ± 21.66	4.17% ± 3.85%	1.56 ± 0.64	12.73% ± 2.49%
PSO	5.84% ± 2.32%	39.44 ± 7.99	1.19% ± 2.00%	0.38 ± 0.38	21.27% ± 9.35%
OOR	2.43% ± 3.33%	46.87 ± 27.29	4.32% ± 5.44%	0.54 ± 0.16	5.67% ± 1.92%
MON	12.91% ± 4.29%	29.08 ± 7.30	1.79% ± 1.42%	0.94 ± 0.28	22.53% ± 7.41%
b					
**Type of maintenance**	**KPI 13** No. of devices per technician		**KPI 14** Time cost of the workforce		
	**–**		(h)		
	Internal maintenance	Service	Internal maintenance	Service	
**CM**	221	137	5412.50	906.35	
**SM**	221	82	2247.70	348.90

**Table 7:** Financial KPIs

Equipment class	Financial KPI					
	KPI 15 COSR (cost of service ratio = global maintenance to acquisition cost) (%)	KPI 16 External maintenance cost (% with respect to total maintenance cost)	KPI 17 Internal maintenance cost (% with respect to total maintenance cost)	KPI 18 Corrective maintenance cost (CM) (% with respect to total maintenance cost)	KPI 19 Scheduled maintenance cost (SM) (% with respect to total maintenance cost)	KPI 20 Cost of spare parts (+ consumables) (% with respect to total maintenance cost)
	Mean 5Y ± SD	Mean 5Y ± SD	Mean 5Y ± SD	Mean 5Y ± SD	Mean 5Y ± SD	Mean 5Y ± SD
CMO	2.31% ± 0.37%	98.71% ± 0.41%	1.19% ± 0.51%	65.02% ± 0.48%	34.88% ± 0.55%	0.10% ± 0.13%
TOP	2.32% ± 0.20%	63.52% ± 14.44%	2.55% ± 1.04%	94.61% ± 3.06%	5.39% ± 3.06%	33.93% ± 13.72%
UTC	2.99% ± 1.56%	65.61 ± 41.17%	34.39% ± 41.17%	59.15% ± 1.91%	31.85% ± 1.03%	9.00% ± 2.94%
ANS	5.30% ± 0.84%	91.00% ± 2.94%	–*	73.91% ± 16.13%	17.44% ± 9.93%	6.79% ± 8.04%
ELB	1.45% ± 0.81%	51.90% ± 0.82%	41.31% ± 15.18%	58.83% ± 2.87%	33.02% ± 2.61%	8.87% ± 4.53%
VPO	8.48% ± 0.35%	91.02% ± 4.62%	0.14% ± 0.19%	63.33% ± 26.73%	31.96% ± 26.41%	4.16% ± 7.55%
ACH	2.30% ± 2.04%	95.30% ± 7.55%	–*	56.73% ± 9.92%	29.42% ± 5.11%	13.77% ± 14.70%
ECG	2.56% ± 0.40%	85.03% ± 15.91%	1.20% ± 0.97%	42.13% ± 16.86%	28.14% ± 12.46%	29.73% ± 13.94%
LSC	2.44% ± 0.87%	4.33% ± 4.53%	65.94% ± 14.12%	27.55% ± 6.39%	10.30% ± 3.18%	62.46% ± 8.25%
DEF	1.60% ± 0.71%	37.85% ± 8.25%	–*	76.96% ± 8.13%	18.05% ± 9.16%	4.96% ± 2.58%
PSO	0.68% ± 0.40%	52.14% ± 8.53%	42.87% ± 8.54%	46.76% ± 26.46%	16.09% ± 7.60%	34.82% ± 27.02%
OOR	4.33% ± 2.49%	20.56% ± 33.76%	43.29% ± 22.53%	64.78% ± 1.32%	29.42% ± 4.37%	1.63% ± 0.96%
MON	3.61% ± 0.81%	96.36% ± 1.68%	2.00% ± 0.83%	65.02% ± 0.48%	34.88% ± 0.55%	0.10% ± 0.13%

*Fully external maintenance

Concerning the economic KPIs, the COSR (*cost of service ratio*) results (i.e. KPI 15) were compared with the values of the economic indicators proposed by the Procurement Unit of the Italian Public Administration CONSIP (i.e. Public Information Services Licensee). The value estimates were in line with the CONSIP values [7]. In this comparison, the electro-surgery unit class deserves further investigation because it has an average COSR value (1.45%) lower than that of CONSIP. Until 2014, there was a no-cost maintenance service policy in place for many of these devices (by contract, the cost was absorbed by the purchase of consumables); this fact strongly influenced this value. In addition, the range of the electro-surgery technology was highly variable, requiring extremely specialized equipment, with high initial purchase costs and very high consumable costs. CONSIP estimates put electro-surgery maintenance incidence at a medium-high level of 8%, so it seems clear that a policy with a service formula would be preferable to purchase.

The results related to KPI 16 (*external maintenance cost*) and KPI 17 (*internal maintenance cost*) for the class of electrocardiographs, which are characterized by both internal and external maintenance policies, should be highlighted due to the impact of this mixed policy on total maintenance costs. From 2012 to 2014, external maintenance costs accounted for an average of 93.03% of the total cost. Beginning in 2015 through 2016, the maintenance policy changed and internal technicians provided maintenance. Therefore, between 2015 and 2016, the impact of external maintenance costs dropped to an average of 69.48% of the total cost. This is an example of how *evidence-based* maintenance works. Because of the experience of internal technicians, starting from the evidence, it was possible to adjust the maintenance policy, leading to economic improvement.

A comparison of the cost patterns for KPI 18 (*CM cost*) and KPI 19 (*SM cost*) on a single class of equipment (telemetry) showed that preventive maintenance accounted for an average of 5.39% of the cost compared with corrective maintenance, which accounted for 94.61%. This difference can be explained by considering the 0.56 average coverage rate of scheduled maintenance for this class. Therefore, to improve this situation, the maintenance policy should be changed to guarantee that preventive maintenance will be performed on each device at least annually. This improvement in the maintenance schedule would probably reduce corrective maintenance costs.

Among the most unusual *cost patterns for spare parts* (KPI 20) were those for surgical tables and pulse oximeters, whose average values were 34.05% and 34.82%, respectively. Indeed, these classes of equipment have accessories and spare parts that wear so rapidly that maintenance is closely linked to their hours of use; they often fail and are replaced. On average, for the other classes, there is a percentage incidence of 30% for SM and a percentage incidence of 60% for CM. The remaining portion is attributable to the costs of the spare parts, affected with higher or lower relevance depending on the type of contract: full risk contracts, for example, include all the spare parts in the annual fee. For equipment with this type of contract, including monitors and monitoring stations, the cost of spare parts, calculated as a separate item, is very low.

Comparison between downtime with no negligent maintenance service and downtime (KPI 1) due *to negligent actions* (KPI 8)—i.e. service interventions lasting more than 30 days—showed that, for all classes, negligent maintenance service led to a considerable increase in downtime (2%, on average), with notable patterns for surgical tables, anaesthesia and telemetry.

**Fig. 3:**
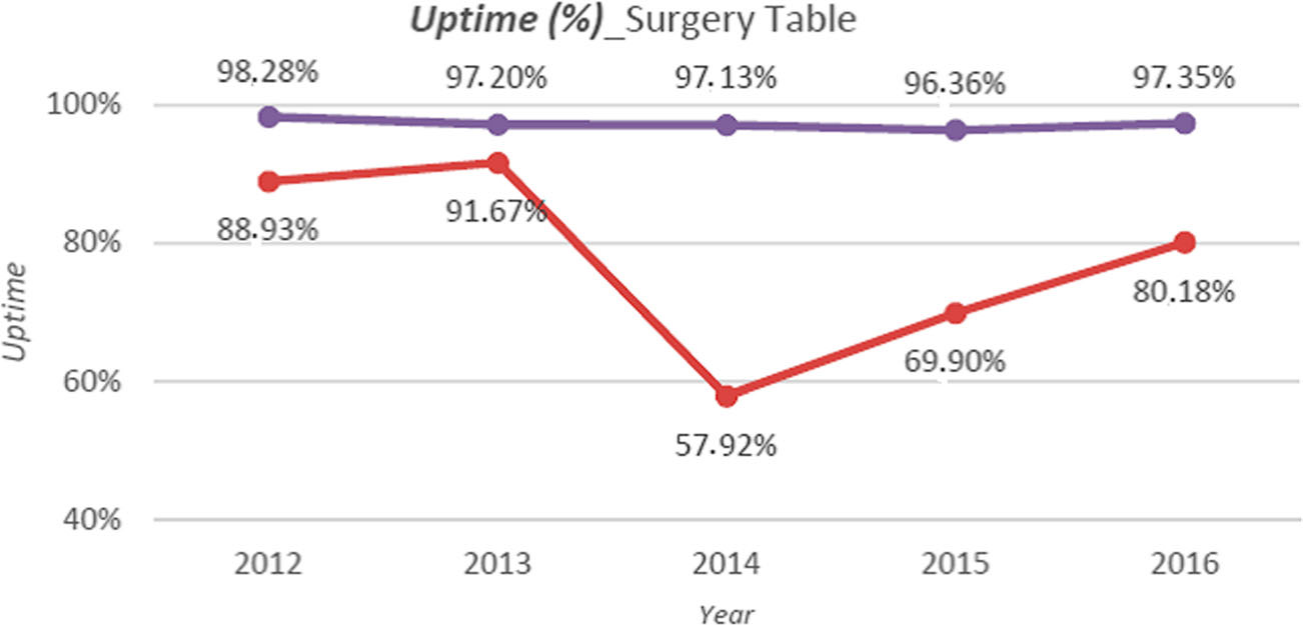
Surgery table: uptime pattern with (red-dashed line) and without (purple line) negligent maintenance service

For the first and the second equipment, downtime was affected significantly by the time required for spare parts to be delivered. Indeed, for these categories, failures of accessories (ACC) were significant. Any equipment downtime directly affects its availability or uptime (KPI 2). The uptime pattern for surgical tables with and without negligent maintenance service is shown in Fig. 3. Clearly, negligent maintenance service significantly affected uptime.

For the anaesthesia machine, it is also necessary to consider that the SM has a long duration for each intervention and, considering that this class has an average SM coverage rate of 1.09, the time dedicated to preventive maintenance affects the availability of the equipment (13.6%, on average) and, consequently, the uptime. Instead, the problem with telemetry could be linked to a higher incidence of negligent maintenance service. This leads to a hope for an organizational correction of the maintenance policy. Specifically, the implementation of an accurate monitoring system for service calls protracted over time, such as a dashboard, could reduce negligent maintenance service that prolongs downtime and delays the availability of the device.

The average annual uptime value, directly related to the downtime figure, for all equipment is better than 94%, which is the CONSIP figure that should be guaranteed each year.

**Fig. 4:**
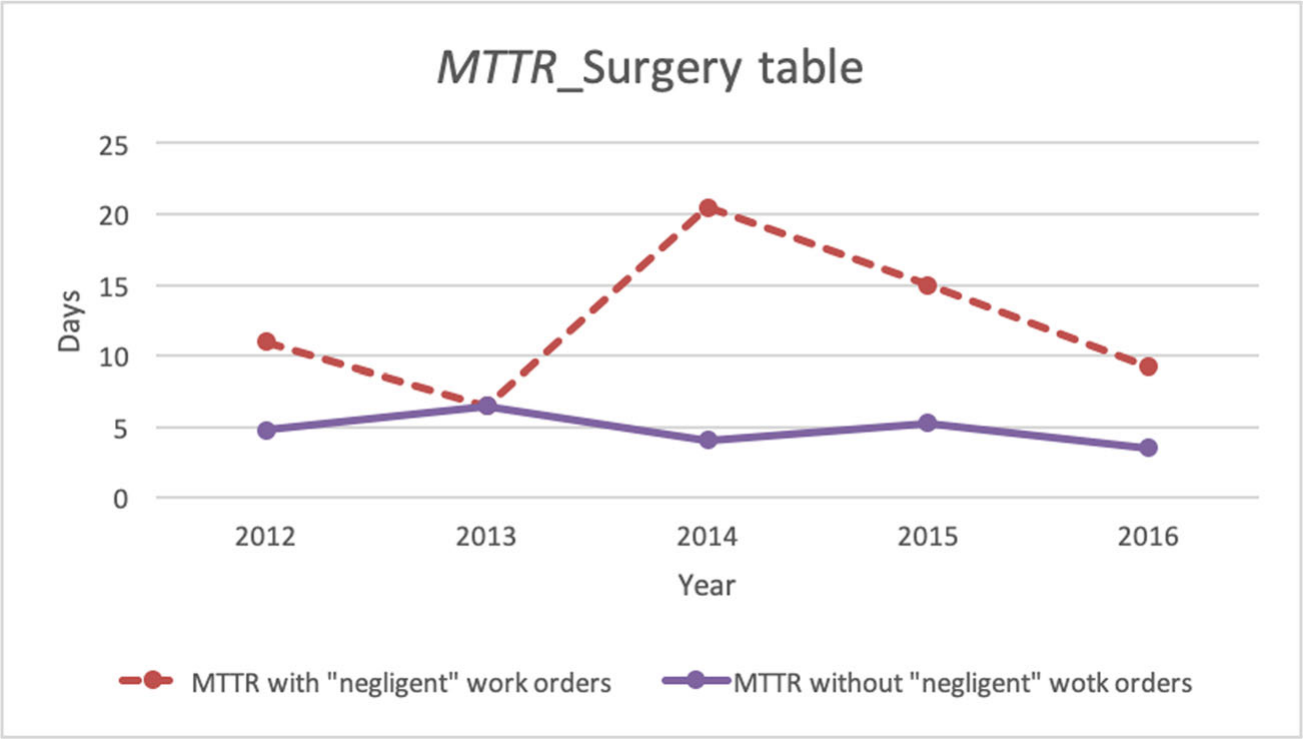
Surgery table: MTTR pattern with (red-dashed line) and without (purple line) negligent maintenance service

It is no surprise then that the class with the highest average MTTR (KPI 3) values (approximately 4.5 days) is “surgical tables,” which, as mentioned above, are affected by negligent maintenance service. The time required to restore the correct function of the devices in this category is strictly linked to the time required for spare parts shipping. The surgical tables’ MTTR pattern in Fig. 4 shows a peak in 2014 for the MTTR with negligent maintenance service. Instead, the MTTR without negligent maintenance service is at the minimum uptime for that year.

The MTBF (KPI 4) should be as high as possible. An acceptable figure is one failure every 6 months (approximately 4500 h). Surgical tables and anaesthesia have the worst (i.e. lowest) values. These values agree with the *global failure rate* (KPI 6) figures. A low global failure rate is generally associated with equipment with low technological complexity. In fact, operating tables and anaesthesia have the lowest MTBF values (1 failure every 2 months), while monitors, pulse oximeters and aspirators have the highest. These values are in agreement with the global failure rate, which is the lowest for these three categories of equipment, while it is greater than 1 (more than one fault per year) for both operating tables and anaesthesia. This result indicates that operating tables and anaesthesia have a greater defectiveness than, for example, pulmonary ventilators (average failure rate of 1.07). The low KPI6 for aspirators is in line with the low technological complexity of these devices and their low impact on maintenance costs. For pulse oximeters and monitors, medium-high classes on the cost of maintenance, a low KPI6 indicates fewer faults in the equipment itself.

The *class failure ratio* index (KPI 5) indicates that pulmonary ventilators and anaesthesia machines are the classes that have most affected the failure rates in operating rooms and intensive care units over the last 5 years. Although the lung ventilators are the class that most greatly affects the total number of faults, compared with their number, they have a lower defectiveness with respect to the anaesthesia machine. In addition, ventilators and anaesthesia are the classes that most affect the cost of maintenance, having average COSRs of 8.48% and 5.30%, respectively. Therefore, when planning maintenance strategies, a balance must be found between two key aspects. On the one hand, costs need to be contained; on the other hand, the criticality of the equipment must be considered. For example, even if anaesthesia machines have a high impact on maintenance costs, these pieces of equipment cannot receive less maintenance because they have a high failure rate and are vital.

Contrary to expectations, an analysis of the *age failure rate* (KPI 7) shows no correlation between failure rate and obsolescence. This could be due to the presence of *no problem found* (KPI 12, i.e. “fake faults”) that do not represent real failures of the equipment and represent a good 19% of the total corrective interventions (684 out of 3581). This category could introduce a distortive component (bias) that prevents a clear interpretation of the indicator performance. The calculation of this indicator was, therefore, repeated by removing the percentage attributable to NPF for some classes, but in general, a correlation between failure rate and age of the device did not appear evident. However, to calculate KPI 7, the equipment was divided into age classes, and this subdivision provided useful information on the age composition of the operating room and intensive care equipment (see Table 7). The data could then be compared with the average age from the literature. For example, among the analysed classes of equipment, it was found that defibrillators, ECGs, aspirators, ventilators and operating tables comprise more than 40% of devices that are older than 10 years. By consulting the data from the Biomedical Engineering Advisory Group (BEAG) [4] and the American Hospital Association (AHA) [26], it can be noted that the average age of operating tables and vacuums is equal to 15 years, that of ventilators and ECG ranges between 7 and 10 years, and that of defibrillators is 5–7 years. Therefore, in our data, defibrillators have an average higher age with respect to the values reported by BEAG and AHA. From the analysis of the failure pattern, however, the classes of equipment in which there is a high percentage of devices over 10 years of age do not always show a higher failure rate than the classes with newer devices.

The age indicator pattern suggests that the variability in age in terms of failure rate is probably much less than the variability of other factors, such as whether operators manage and use the devices properly or not. Therefore, age does not seem to be a significant parameter in current maintenance policies.

*Negligent maintenance service* (KPI 8) (corrective maintenance calls resolved in more than 30 days) are to be applied together with uptime (KPI 2), downtime (KPI 1) and MTTR (KPI 3). Compared with the total number of failures, the telemetry equipment and surgical table classes have the highest number of negligent maintenance service calls. Instead, the number of *1-day service calls* (KPI 9) (corrective maintenance service requests resolved within 24 h) was found to be lower for telemetry equipment, which were more affected by negligent maintenance service. However, 64.49% of defibrillators were serviced within 24 h. This equipment also has KPI 1 and KPI 3.

No problem found (KPI 12) was more significant for aspirators and anaesthesia machines. However, if compared with the involved workload (approximately 5 days a year), it does not greatly affect the wasted time. In this case, investing in training to instruct staff to open corrective interventions in a better way would not be economically advantageous because the incidence of these “fake faults” does not justify the investment.

The SM coverage rate (KPI 11) is higher than 1 SM intervention per year for surgical lamps, ventilators and anaesthesia machines. For defibrillators, only in 2016 is the planned target of 2 preventive maintenance interventions per year reached because of the transition from internal service to external global service, due to a low internal service coverage rate in previous years. Other classes of equipment have a value of KPI 11 less than 1 mainly because devices that cannot be found or that are used continuously are unavailable for maintenance activities. Ceiling-mounted units from 2012 to 2015 have KPI 11 values less than 0.4. This low value is due to an SM frequency of 2 years, as specified in technical manuals. In Table 8, the yearly SM coverage rates for each equipment class are shown from 2012 to 2016.

**Table 8:** SM coverage rate

Equipment class			KPI 11–SM coverage rate		
	2012	2013	2014	2015	2016
CMO	0.71	0.59	0.67	0.63	0.77
TOP	1.10	0.43	0.69	0.87	1.14
UTC	0.50	0.95	0.40	0.16	0.79
ANS	1.01	0.99	0.95	1.30	1.18
ELB	0.87	0.80	0.93	0.57	0.68
VPO	1.14	1.35	1.30	1.11	1.63
ACH	0.05	0.23	0.38	0.14	0.71
ECG	0.53	0.61	0.58	0.40	0.45
LSC	0.48	1.05	1.32	1.17	1.89
DEF	0.97	1.56	1.48	1.17	2.63
PSO	0.30	0.25	0.32	0.37	0.68
OOR	0.44	0.73	0.32	0.56	0.65
MON	0.71	0.61	0.99	1.13	1.26

By comparing internal maintenance and external service in terms of *number of devices per technician* (KPI 13) and *time cost of the workforce* (KPI 14) in CM and SM, it can be concluded that, in relation to the considered equipment, the internal technicians manage a higher number of devices than the service at the expense of more hours spent in maintenance. Despite a higher workload, the internal technicians mainly manage classes of equipment such as the operating room cabinets, telemetries and scialytic lamps. They are highly specialized in these types of equipment; hence, they are able to optimize their timing and manage a greater workload.

The *SM with failures* (KPI 10) shows that, on average, SM does not lead to the detection of failures (in fact, the mean value for the 5 years is less than 12%). Therefore, SM does not always succeed in intercepting failures or problems. Based on what has been discussed so far, to improve or correct the maintenance policy, it may be assumed that one ought to start with the evidence (i.e. the failure data). Planning a certain maintenance strategy is not enough. Instead, it is necessary to continuously monitor the behaviour of the key parameters with the greatest impact on equipment availability (uptime, negligent maintenance service calls, MTTR). This will not only optimize available resources but also improve the effectiveness of the maintenance service and ultimately improve patient and operator safety.

### Combined use of the maintenance service codes and KPI

The combined use of the two approaches discussed thus far provides a tool for broad spectrum monitoring of the maintenance process. Indeed, the KPI values can be better investigated and understood by making use of the types of coded maintenance calls. Similarly, if an unusually high failure type is detected, the effects of this problem on the performance of the entire maintenance process may be observed. In this way, targeted corrective actions can be taken to improve maintenance service.

For example, if surgical tables have above-average downtime and MTTR, this can be investigated in greater depth by analysing the evidence, or rather, the types of failures. The spare parts cost indicator also shows a high impact on total maintenance costs for the surgical tables. From an analysis of the coded maintenance calls, one can find, that for this category of equipment, accessory failures have a greater incidence. The actions taken to improve the process can include monitoring the time needed for spare parts shipping and taking a survey to identify the most failure-prone accessories (models and brands) for consideration during procurement.

For example, on average, 34.39% of costs were for internal telemetry maintenance. More than half of all maintenance costs were for repairs that required sending the device back to the company for service. For this equipment class, the influence of negligent maintenance service (maintenance calls that last more than 30 days) affects not only equipment availability, which is a prolonged period out of service, but also maintenance costs, which represent the greater part of the cost items.

Another analysis procedure could be performed beginning with the classified service calls. Through a review of the categories with unusually high failure, the reasons for this singular behaviour may be investigated in more detail using KPIs. For example, electro-surgery units showed a peak of component failures in 2016. An analysis of the coverage rate indicator for scheduled maintenance calls showed that, in 2015 and 2016, SM coverage was not optimal. In addition, the analysis of the coded corrective maintenance calls in 2016 showed that 100% of the calls were classified as NPF. This seems to suggest that specific checks on the components that tend to fail most during scheduled maintenance calls should be included.

Another example concerns the defibrillators, where an analysis of the fault types revealed a prevalence of battery failures (BATT). An analysis of the scheduled maintenance indicator showed poor coverage for defibrillators, which improved in 2016, the year the equipment was transferred to a global service policy.

However, in 2016, the global failure rate for the defibrillator class was the highest of the 5 years analysed. This suggested that, despite adequate scheduled maintenance coverage, it might be useful to include specific battery status checks during the scheduled inspections. Given that 67% of the total maintenance costs for this class of equipment were represented by battery costs, appropriate battery management could also reduce maintenance costs.

## Discussion

By analysing the classes of equipment with the fault codes proposed in this study, it is possible to understand the types of problems most frequently encountered, which could be useful for longitudinal and transversal comparisons. Longitudinal comparisons involve the analysis of a certain maintenance policy at a given hospital before and after the adoption or modification of a certain strategy. Starting from the adoption of the *evidence-based* approach, the hospital could monitor the changes over time and compare them with the results of the previous maintenance policy. Transversal comparisons examine other hospital circumstances.

The classification of maintenance service calls has highlighted that the main problem to be overcome is the need for descriptions of the maintenance work done that are as accurate as possible. To a certain extent, by using failure codes, it may be possible to be freed of the need for detailed descriptions of the maintenance service calls to track the specific type of failure or problem found.

By introducing the classification and analysis of maintenance service calls as a part of the daily duties of maintenance technicians, a valuable new tool that characterizes the equipment classes in terms of problems and failures found can be implemented. The implementation of this approach could very well lead to greater optimization of the use of human and technological resources.

It should be noted that the adoption of the identified performance indicators provides a dual function in terms of the assessment and control of maintenance process performance and data communication and sharing. The data and information conveyed by a hospital dashboard can provide summarized yet complete output documents, which can represent objective support for upper management decisions.

The set of performance indicators defined concerned technological, organizational and financial aspects. The problems encountered with the technology indicators were related to scheduling. In fact, the data had to be updated in real time to enable the assessment of the actual availability of equipment and troubleshooting times.

The problems with the organizational and financial indicators were similar. Essentially, these consist of the lack of a single source for the data. To implement a set of indicators displayed in a hospital dashboard, the indicators must be updated from a single data source. However, the fact that a complete financial analysis requires the consultation of several sources, which belong to structurally different processes and therefore cannot be merged into a single database, should also be considered.

## Conclusion

Maintenance is a crucial aspect of the activities in a hospital’s CE department because it involves significant human and financial resources. The assessment of the effectiveness of any maintenance programme is fundamental to the optimization of the use of available resources in CE departments. In this respect, an approach for monitoring maintenance from multiple points of view can be implemented by combining the classification of maintenance service calls and key performance indicators. This in turn enables targeted service to be performed, which can be applied to the assessment and modification of maintenance strategies and policies. In fact, scheduled maintenance cannot always intercept problems before they arise. Maintenance processes must be continuously monitored, starting with the failures, so that the issues that most affect maintenance effectiveness can be identified and managed.

Data of the fault classification and KPIs were uploaded to the business intelligence (BI) software QlikView® to obtain a global view of all equipment classes and results. Specifically, four types of worksheets were conceived: one worksheet is related to maintenance policy and types of faults, and the other is related to costs, technological KPIs and corresponding maintenance times. A study is currently being carried out to test the use of most recent BI open source software solutions, such as Apache Superset.

In this way, strategies can be modified so that an appropriate compromise between the criticality of the equipment and the need to contain costs may be achieved.

Clinical engineering departments can promote failure reduction by adopting strategies of training (to reduce errors related to use and maintenance interventions with “no problem found”). They can also prepare tender specifications based upon the evidence from these KPIs. Finally, they can design new protocols for internal maintenance based upon this analysis.

A new management software, currently being developed at a regional level, is implementing the innovative set of KPIs proposed in this work. Its commissioning will provide all the public hospitals in Tuscany with a great opportunity to apply the principles of evidence-based maintenance and will create the opportunity to compare different hospitals in future works.
